# Improving GLONASS Precise Orbit Determination through Data Connection

**DOI:** 10.3390/s151229790

**Published:** 2015-12-02

**Authors:** Yang Liu, Maorong Ge, Chuang Shi, Yidong Lou, Jens Wickert, Harald Schuh

**Affiliations:** 1 GNSS Research Center, Wuhan University, 129 Luoyu Road, Wuhan 430079, China; liudaweng@126.com (Y.L.); shi@whu.edu.cn (C.S.); 2 German Research Center for Geosciences (GFZ), Telegrafenberg, Potsdam 14473, Germany; maor@gfz-potsdam.de (M.G.); wickert@gfz-potsdam.de (J.W.); schuh@gfz-potsdam.de (H.S.)

**Keywords:** GLONASS, data connection, precise orbit determination, orbit precision

## Abstract

In order to improve the precision of GLONASS orbits, this paper presents a method to connect the data segments of a single station-satellite pair to increase the observation continuity and, consequently, the strength of the precise orbit determination (POD) solution. In this method, for each GLONASS station-satellite pair, the wide-lane ambiguities derived from the Melbourne–Wübbena combination are statistically tested and corrected for phase integer offsets and then the same is carried out for the narrow-lane ambiguities calculated from the POD solution. An experimental validation was carried out using one-month GNSS data of a global network with 175 IGS stations. The result shows that, on average, 27.1% of the GLONASS station-satellite pairs with multiple data segments could be connected to a single long observation arc and, thus, only one ambiguity parameter was estimated. Using the connected data, the GLONASS orbit overlapping RMS at the day boundaries could be reduced by 19.2% in ideal cases with an averaged reduction of about 6.3%.

## 1. Introduction

GLONASS is currently another Global Navigation Satellite System (GNSS) with full operational capability other than GPS. The precise orbit determination (POD) is the prerequisite for numerous GNSS applications with the demand of high accuracy. Since 1998, the GLONASS precise orbit products were developed within the International GLONASS Experiment (IGEX) [1]. From 2001, the International GNSS Service (IGS) [2] established the International GLONASS Service Pilot Project (IGLOS-PP) [3]. Through these efforts POD of the GLONASS constellation has been gradually improved [4,5,6,7] and its orbit precision currently reaches the centimeter level. However, it is still markedly worse than that of GPS in terms of the orbit agreement among different IGS analysis centers and over-day boundaries [8,9,10]. One of the major reasons is that the GPS solution is very much strengthened by its successful ambiguity resolution even over baselines up to several thousand kilometers [11,12,13,14]. Unlike GPS, which adopts code division multiple access (CDMA), the present GLONASS uses frequency division multiple access (FDMA) to distinguish the signals from individual satellites [15]. It introduces different hardware biases to each receiving channel, thus makes its ambiguity resolution rather difficult, especially for long baselines. 

In order to increase the strength of the GLONASS solution, several studies on the GLONASS ambiguity resolution have been carried out. Most of the developed mathematical and stochastical modeling methodologies and specific ambiguity resolution strategies are systematically summarized and investigated by Wang *et al.* [16]. It is well known that through the calibration of carrier-phase inter-frequency biases at the GLONASS receiver-end, the ambiguity resolution for baselines of several hundred kilometers can be achieved [17]. In the IGS data processing at CODE (Center for Orbit Determination in Europe), for baselines shorter than 200 km, the GLONASS ambiguity resolution is enabled for all satellites, whereas for longer baselines only ambiguities of the same frequency channels are considered in order to improve the orbit quality [18]. However, most of the IGS analysis centers have not yet implemented the GLONASS ambiguity resolution most likely due to its complexity and limited benefit.

It is quite meaningful to further improve the accuracy of GLONASS orbit for high-precision GNSS applications. A novel method, *i.e.*, data connection rather than ambiguity resolution, is introduced and investigated in this contribution. As demonstrated in [19], when using carrier ranges [20] generated with the undifferenced integer ambiguities in precise point positioning (PPP) [21,22] for single stations, the data continuity for GPS satellites is increased and the averaged RMS of the overlapping orbits is reduced. It is also demonstrated that most observation segments, even that from separated satellite passes with a data gap of hours, can be connected. Inspired by this fact, we present a method for data connection to improve GLONASS orbit quality by using the connected data. Since currently the GLONASS PPP ambiguity resolution is rather difficult to be realized on a global scale, instead of using the carrier-range method, the data are connected by checking the consistency of the wide-lane and narrow-lane ambiguities of different observation segments. After the successful data connection, GLONASS orbit quality is expected to be improved. In this contribution, we try to make a thorough performance assessment of the data connection method and analyze possible benefits for GLONASS orbit determination.

The data connection method is presented in the next section. Then experimental validation is described with the details of the network, data set and processing strategy. The performance of the data connection and the improvement on GLONASS orbits in terms of overlapping RMS are presented and investigated in subsequent sections, followed by conclusions and suggestions.

## 2. Data Connection Method 

In general, we assume that undifferenced ionosphere-free phase ($L_{c}$) and range ($P_{c}$) observations are used in the data processing, and the observation equations are:

(1) $$\begin{array}{l} L_{c}=\frac{f_{1}^{2}}{f_{1}^{2}-f_{2}^{2}}L_{1}-\frac{f_{2}^{2}}{f_{1}^{2}-f_{2}^{2}}L_{2}=\rho +dt_{R}^{S}+\lambda_{1}b_{c}\\ P_{c}=\frac{f_{1}^{2}}{f_{1}^{2}-f_{2}^{2}}P_{1}-\frac{f_{2}^{2}}{f_{1}^{2}-f_{2}^{2}}P_{2}=\rho +dt_{R}^{S} \end{array}$$

where $L_{1}$, $L_{2}$, $P_{1}$, and $P_{2}$ are phase and range observations of the two frequencies $f_{1}$ and $f_{2}$ in the unit of length, respectively. $dt_{R}^{S}$ is the receiver clock bias minus satellite clock bias. ρ is the non-dispersive delay including the geometric distance, the tropospheric delay, and any other delay which affects the observations identically, with the correction of the phase center [23], the phase windup effect [24], and the satellite-dependent differential code biases (DCB) [25]. $b_{c}$ is the $L_{c}$ ambiguity. Multipath effect, hardware delay, and noise are not included in the equation for clarity. 

For the ambiguity resolution, ambiguity $b_{c}$ is usually expressed by wide-lane (WL) and narrow-lane (NL) ambiguities [12] as:

(2) $$b_{c}=\frac{f_{1}f_{2}}{f_{1}^{2}-f_{2}^{2}}b_{w}+\frac{f_{1}}{f_{1}+f_{2}}b_{n}$$

Usually, WL ambiguities are fixed based on the Melbourne–Wübbena (MW) combination [26,27], whereas NL ambiguities are derived based on the fixed WL and the estimated ionosphere-free ambiguities and fixed accordingly. 

We follow the procedure of the ambiguity resolution to connect the WL ambiguities first, and then the NL ambiguities. Without loss of generality, we assume that there are n observation segments for a station-satellite pair and their WL ambiguities and the corresponding standard deviations computed using the MW combination are ${\left[b_{wi},\sigma_{wi}\right]}_{i=1,2,...,n}$. For the observation segments j and k, we can get the difference and its standard deviation as:

(3) $$\begin{array}{l} b_{wjk}=b_{wj}-b_{wk}\\ \sigma_{wjk}=\sqrt{\sigma_{wj}^{2}+\sigma_{wk}^{2}} \end{array}$$

If there is a unique integer value $I_{w}$ and it satisfies:

(4) $$I_{w}-3\sigma_{wjk}<b_{wjk}<I_{w}+3\sigma_{wjk}$$

then WL of the two segments can be connected by applying the phase integer offset of $I_{w}$. Obviously, the standard error of the difference, *i.e.*, $\sigma_{wjk}$ must be small enough in order to obtain a unique phase integer offset. 

A more sophisticated way to make the decision whether the two WL ambiguities can be connected is to use the following probability function [12]:

(5) $$P=1-\sum_{n=1}^{\infty }\left[erfc(\frac{n-(b_{wjk}-I)}{\sqrt{2}\sigma_{wjk}})-erfc(\frac{n+(b_{wjk}-I)}{\sqrt{2}\sigma_{wjk}})\right]$$

(6) $$erfc(x)=\frac{2}{\sqrt{\pi }}\int_{x}^{\infty }e^{-t^{2}}dt$$

where I is the integer candidate for $b_{wjk}$ to be tested. 

Taking the confidence level α of data connection as 0.1%, if P is bigger than 1−α, the WL ambiguities of the two segments can be connected confidently with the phase integer offset $I_{w}=I$, otherwise they cannot be connected. 

After the WL ambiguities are connected with an phase integer offset $I_{w}$, *i.e.*, $b_{wj}=b_{wk}+I_{w}$, we can get the corresponding NL ambiguity with the connected WL ambiguities and the ionosphere-free ambiguities estimated from the adjustment as:

(7) $$b_{nk}=\frac{f_{1}+f_{2}}{f_{1}}b_{ck}-\frac{f_{2}}{f_{1}-f_{2}}b_{wk}$$

(8) $$b_{nj}=\frac{f_{1}+f_{2}}{f_{1}}b_{cj}-\frac{f_{2}}{f_{1}-f_{2}}(b_{wk}+I_{w})$$

Similar to the wide-lane, their difference and the corresponding standard deviation are:

(9) $$\begin{array}{l} b_{njk}=b_{nj}-b_{nk}\\ \sigma_{njk}=\frac{f_{1}+f_{2}}{f_{2}}\sqrt{\sigma_{cj}^{2}+\sigma_{ck}^{2}-2\sigma_{cjk}} \end{array}$$

It should be pointed out that the correlation term in Equation (9) could not be ignored, as the STD of the two undifferenced ambiguities from the adjustment are usually rather large. Moreover, for the sake of computational efficiency, the covariance of ambiguities is not available, in this case, a default value of 0.05 cycles can be applied at least for processing data of long segments from our expertise in the previous studies on ambiguity resolution [28] as well as in this research. 

Similar to the WL connection, we can determine whether the two NL ambiguities can be connected according to the probability Equation (5). Based on the approximation, if the fractional parts of the two NL ambiguities are close to each other, with the difference of 0.15 cycles, the two NL ambiguities can be connected. Let the corresponding phase integer offset be $I_{n}$, we have $b_{nj}=b_{nk}+I_{n}$. It is worth mentioning that two observation segments of a station-satellite pair are regarded as connected, only when both the WL and NL ambiguities are connected.

By considering that $b_{w}=b_{1}-b_{2}$ and $b_{n}=b_{1}$, we can get the relationship between the L1 and L2 ambiguities of the two segments, respectively, as:

(10) $$\begin{array}{l} \left\{ \begin{array}{l} b_{1k}=b_{nk}\\ b_{2k}=b_{nk}-b_{wk} \end{array}\right.\\ \left\{ \begin{array}{l} b_{1j}=b_{nk}+I_{n}\\ b_{2j}=b_{nk}-b_{wk}+(I_{n}-I_{w}) \end{array}\right. \end{array}$$

This can also be expressed by the phase integer offsets between the observation segments k and j in the L1 and L2 frequencies as $N_{1}$ and $N_{2}$, with $N_{1}=I_{n}$, and $N_{2}=I_{n}-I_{w}$. Applying the phase integer offsets to the second segment, these two segments are connected and only the ambiguity at the data beginning is remaining.

Performing this data connection procedure for all the observation segments of a station-satellite pair iteratively until no new segments can be connected any more. After successful connection, the estimates of the connected segment can be updated for further processing. It should be mentioned that the data connection method can be applied to GLONASS FDMA signals, as well as to CDMA signals adopted by the other constellations, as the connection is carried out for each station-satellite pair.

As to the implementation issue, the phase observations are corrected with the determined phase integer offsets and for each station a new RINEX observation file is generated with a special flag indicating the corrected phase integer offsets. With such corrected observation files, the estimation processing is carried out in the same way as usual except that no ambiguity is estimated for the connected segments. In practice, careful screening of the post-fit residuals should be implemented to confirm the correctness of the connection and to remove possible incorrect connections.

## 3. Experimental Validation

**Figure 1:**
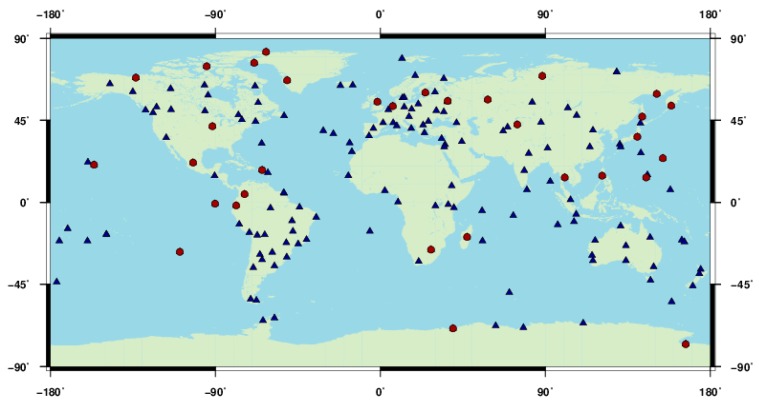
Geographical distribution of the 175 IGS tracking stations used in the experiment, dots are for GPS-only stations and triangles for GPS/GLONASS stations.

In order to validate the connection method described above, an experiment was carried out for data connection in GLONASS POD. We adopted combined orbit determination of GPS and GLONASS on the observation level using globally well distributed IGS stations with daily observations, as the combination with GPS can improve the GLONASS satellite orbits [8]. Observations of 175 IGS stations from DOY 270 to 300, 2013 were used. Figure 1 shows the geographical distribution of the tracking stations, where 32 GPS-only stations are represented by red dots and the rest GPS/GLONASS stations by blue triangles.

The Positioning and Navigation Data Analyst (PANDA) [29,30] software was adapted and used for data connection and POD processing in this study. In the combined GPS/GLONASS processing, the inter-frequency bias (IFB) in a receiver was estimated as a constant for each GLONASS frequency. Observation segments shorter than 40 min were deleted. Ambiguity resolution for baselines shorter than 3500 km was carried out for GPS using the method in [14] whereas no ambiguity resolution for GLONASS. In the GLONASS data connection, no connection was undertaken if the distance between the ambiguity difference and its closest integer was larger than 0.15 cycles. The POD solution was carried out in accordance with the routine processing at the GFZ (German Research Center for Geosciences) IGS Analysis Center. The other important options of processing strategy about observation model and force model in the experiment are listed in Table 1.

**Table 1:** Observation and force models.

Basic observable	Undifferenced ionosphere-free code and phase combination
Sampling rate	300 s
Arc length	1 day
Cutoff elevation	7°
Weighting	*Priori* precision 0.01 cycle and 0.6 m for raw phase and code, respectively Elevation-dependent, 1 for E > 30°, otherwise 2*sin(E)
Phase center correction	PCO (Phase Center Offset) and PCV (Phase Center Variation) for GPS, GLONASS satellites and stations, igs08.atx [23]
Phase wind up	Corrected [24]
Tropospheric delay	GMF (Global Mapping Function) [31], priori delay [32], 2-hourly ZTD (Zenith Tropospheric Delay), 24-hourly gradients [33]
Clock error	White noise
Earth Rotation Parameters	IERS (International Earth Rotation and Reference Systems Service) C04 [34] tight constraint
Tide displacement	IERS Conventions 2010 [35], FES2004 [36]
Relativity effect	IERS Conventions 2010
Earth gravity	EIGEN_GL04C [37] up to 12 × 12
N-body gravitation	Sun, Moon, Mercury, Venus, Mars, Jupiter, Saturn, Uranus, Neptune, Pluto regarded as point masses, JPL Planetary Ephemeris DE405
Solar radiation	Reduced Empirical CODE Orbit Model (ECOM) five parameters without a background model [38]
Attitude model	GPS model [39], GLONASS model [40]
Earth radiation and antenna thrust	Effects acting on the Box-Wing model applied [41]
Additional empirical acceleration	Constant and periodic once-per--revolution accelerations along-track not estimated

We first analyzed the data connection effectiveness for all the stations, for different receiver types, and then for each GLONASS satellite. Afterwards, GLONASS orbits before and after the data connection were compared with the IGS final orbits. Then, the overlapping RMS of the solution on the day boundaries was assessed to confirm the possible improvements of GLONASS orbit quality. 

### 3.1. Data Connection Ratio

To assess the effectiveness of the data connection, we define an empirical connection ratio, *i.e.*, the number of GLONASS station-satellite pairs that are successfully connected to only one arc divided by the number of GLONASS station-satellite pairs that originally have various observation segments. In the validation experiment, typically a station could track all the 24 GLONASS satellites on a daily basis, where the majority GLONASS satellites had more than one observation segments. Taking the average for all the stations, there were 17 GLONASS satellites that had multiple observation segments in the daily observing duration. The number was reduced to 12.4 on average after the proposed data connection, *i.e.*, 4.6 extra GLONASS satellites were connected to have only one observation arc. Specifically for the WL connection, there were 11.3 extra satellites that were connected to only one arc and the connection ratio was 66.5%. For the NL connection, there were 4.6 extra satellites connected to only one arc and the connection ratio was 27.1%. Obviously, the NL is more difficult to be connected because it has a much shorter wavelength of about 11 cm compared with the WL of about 84 cm. 

On average, there were about 44 ambiguity parameters per station before the data connection and the number was reduced to 39 after the data connection. For all the stations in the daily solution, the total number of GLONASS ambiguities was 6257 and it was reduced to 5546 after the data connection with a reduction percentage of 11.4%. 

**Figure 2:**
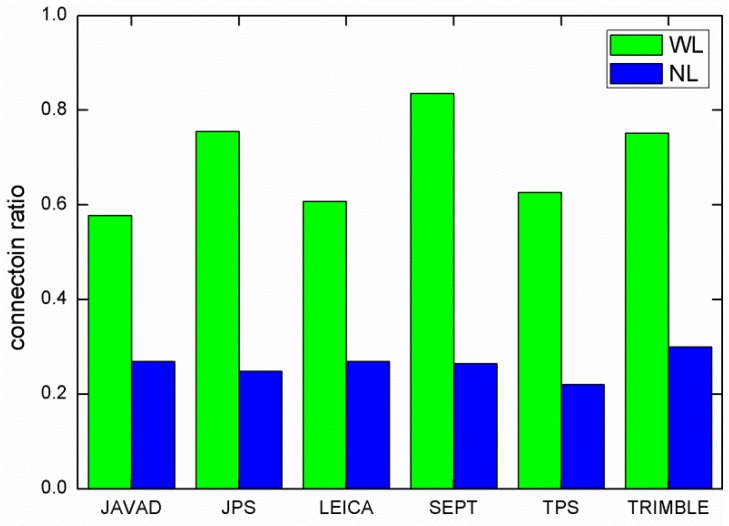
Wide-lane and narrow-lane connection ratio for different type of receivers.

The connection ratios of each different receiver type listed in Table 2 were calculated and are shown in Figure 2 for WL and NL ambiguities, respectively. Since the WL ambiguities are computed using the MW combination, it is only influenced by the measurement noises including carrier phase and code multipath. Therefore, the WL connection ratio among individual receiver types should be attributed mainly to the measurement quality of receiver types. For the NL connection ratio, there was almost no significant difference among individual receiver types, and the connection ratio was rather low of about 26.2%. It is easy to understand that the NL connection is limited by the quality of the solution from which NL ambiguity is derived. This also means that the estimation of the ionosphere-free ambiguity must be further improved in order to obtain a better connection result. 

**Table 2:** GLONASS tracking receiver types and numbers used in the experiment.

Manufacturer	Receiver Number
JAVAD	22
JPS	12
LEICA	43
SEPT	5
TPS	16
TRIMBLE	44

**Figure 3:**
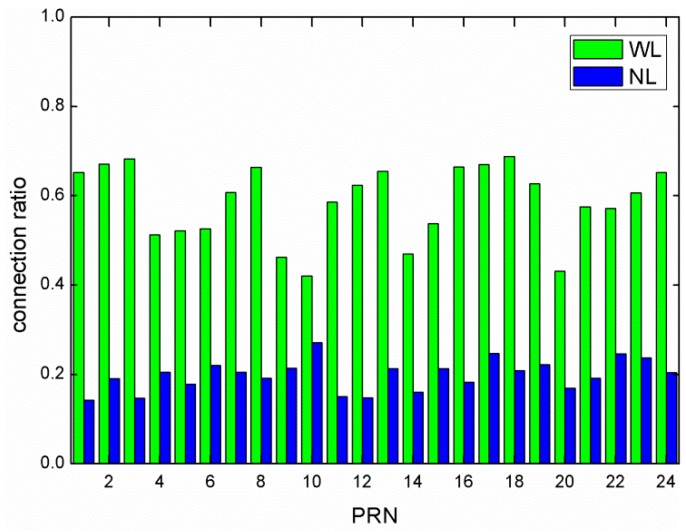
Wide-lane and narrow-lane connection ratio for individual GLONASS satellites.

The connection ratios for individual GLONASS satellites were also calculated and are shown in Figure 3 for WL and NL ambiguities, respectively. Slight differences can be found among individual GLONASS satellites, which are more pronounced for WL connection than for NL connection. For the WL connection, we attribute the differences to the measurement noises and the stability of individual satellite hardware delays, as no other factors seem plausible. For the NL connection, the differences are rather small and could be caused by the different tracking geometry and inaccurate modeling. It should be mentioned that the undifferenced ambiguities are affected by a combination of code and phase hardware delays in the satellite and the receiver [42]. The possible temporal changes in the satellite or the receiver hardware delays can be one error source affecting the success of the data connection method. For example, the long-term temporal variation in the receiver DCB is found to be linearly correlated with the ambient outdoor temperatures [43]. The impact on the daily orbit solutions may also affect the data connection ratio.

### 3.2. Improvement on GLONASS Satellite Orbits

**Figure 4:**
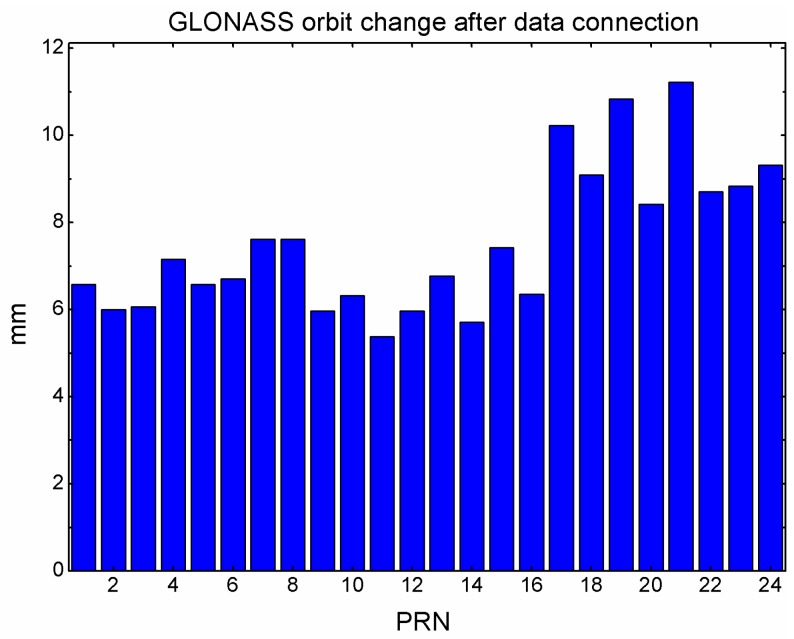
GLONASS satellite orbit variation after the data connection.

In order to show the influence of the data connection, the GLONASS satellite orbits are compared before and after the data connection. The individual GLONASS satellite orbit variation is computed every five minutes in each day and averaged in RMS during the experiment period. The result is shown in Figure 4. The orbit variation is 7.5 mm on average for all the GLONASS satellites. The magnitude of the orbit variation is considerable taking into account that the connection ratio is relatively low and the connection is carried out on a single station basis without strong constraints among different stations. 

**Figure 5:**
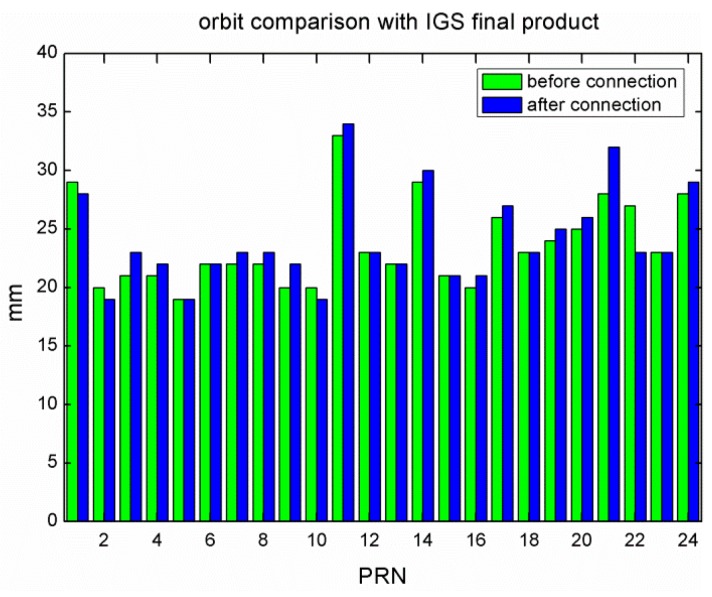
Comparison of GLONASS orbits before and after the data connection with IGS final products.

We first compared the GLONASS satellite orbits before and after the data connection with the IGS final GLONASS orbits. After a seven-parameter Helmert transformation for removing possible systematic differences, the median RMS during the experimental period is shown in Figure 5. The RMS of all the GLONASS satellites was almost the same of 23.7 mm for the two solutions. We can also see that for many GLONASS satellites, the RMS got slightly larger of about 1 mm after the data connection. There was no significant difference between the two solutions, *i.e.*, the orbits were not dramatically improved by the data connection method from the point of orbit differences with IGS final products. It may indicate that it is difficult using the IGS final products to assess the possible orbit improvement of the data connection, although they are of high quality. 

**Figure 6:**
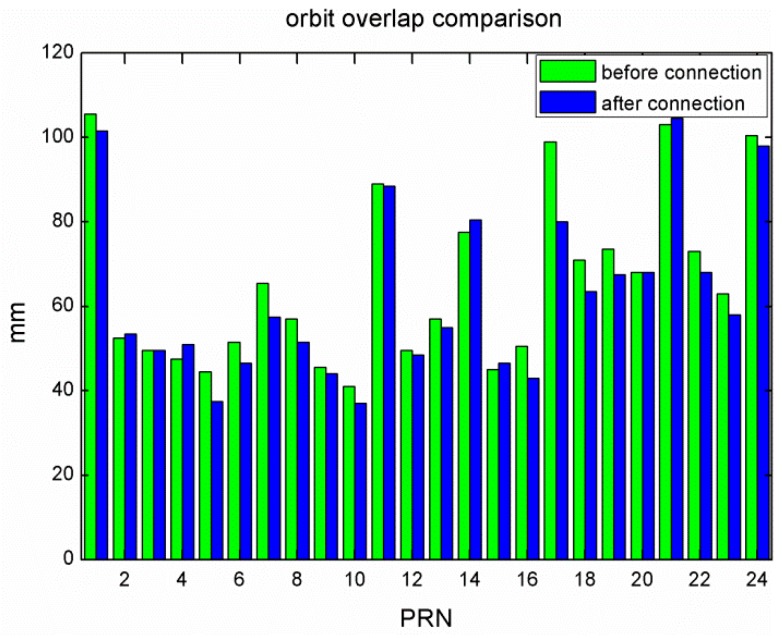
Overlapping orbit RMS for the solution before (green) and after data connection (blue).

For more realistic orbit quality assessment, the discontinuity at the day boundary is adopted as a measure in Griffiths and Ray [9]. The overlapping orbit RMS of the GLONASS satellites before and after the data connection were calculated and plotted in Figure 6. Since the overlapping RMS involve orbits from two consecutive days, the inferred orbit precision for a single daily solution should be divided by $\sqrt{2}$.

Usually, the median RMS of all the satellites is selected as an indicator for the orbit quality because it can avoid the effect of bad satellites with very large RMS [8]. The median RMS before the data connection was 60.0 mm and it was reduced to 56.2 mm by the data connection. The overall improvement was about 6.3%. For individual satellites, the largest improvement was by 19.2% while the smallest improvement was by 0.6%. Thus, data connection can indeed increase the solution strength and improve the orbit quality, although the improvement is not as strong as that of ambiguity resolution which substantially improves the orbit quality for GPS satellites. For comparison, the overlapping orbit RMS of all the GPS satellites was improved by about 30.5% after ambiguity resolution for the experiment period. From Figure 6, five satellites show slight degradation rather than improvement. The possible correlations with the orbital plane, the satellite type, and launch time were checked but no obvious correlation was found, which should be further investigated. Nevertheless, data connection could improve the overall GLONASS orbit quality, before the GLONASS ambiguity resolution for long baselines up to several thousands of kilometers can be implemented reliably.

## 4. Conclusions and Suggestions

Since the reliable GLONASS ambiguity resolution over long baselines is not yet available, we developed a method for data connection to increase the strength of global network solutions. The WL ambiguities of all segments are derived from the MW combination and their NL are calculated based on the connected WL and the estimated ambiguities in the adjustment. The WL and NL ambiguities of each GLONASS station-satellite pair are statistically tested and corrected for phase integer offsets. In this way, GLONASS station-satellite pairs with multiple data segments can be connected and even to a single observation arc, *i.e.*, only one ambiguity parameter is remaining. Since the number of ambiguities is reduced, the strength of the solution will be increased and consequently the orbit quality is improved with the connected data. 

The connection method and its impact on satellite orbits were investigated through an experimental validation using a global network of IGS ground tracing stations. The effectiveness of the connection method was investigated for different receiver types and individual GLONASS satellites. The statistics show that on average 27.1% of the GLONASS station-satellite pairs with multiple data segments can be connected to a single observation arc. The major restriction is in the NL connection most likely due to inaccurate modeling in the estimation. 

The RMS of overlapping orbits for the solutions with and without the data connection were computed and analyzed. The median RMS of the overlapping orbits for all the GLONASS satellites was reduced from 60.0 mm to 56.2 mm after the data connection, which corresponds to an averaged improvement of about 6.3% over all satellites. Furthermore, the largest improvement was up to 19.2% for a single satellite. As the GLONASS ambiguity resolution is not reliably available, the data connection provides an alternative way to improve the orbit quality, although it is not as significant as the ambiguity resolution for GPS.

Finally, from the results of one-month POD experiment, we could conclude that the quality of GLONASS satellites orbits could be slightly improved by the data connection method. The improvement could be increased by more accurate modeling in the adjustment, like the solar radiation pressure model. The recent developments of GLONASS orbit modeling, e.g., by improving the empirical ECOM model [44] or applying the adjustable box-wing model [45], should be implemented and investigated in the future. Furthermore, the connection can still be utilized to improve the solution even if the reliable GLONASS ambiguity resolution is implemented. 
